# Anglo-American Dental Depot

**Published:** 1858-01

**Authors:** J. Ravenscroft

**Affiliations:** 10 & 11 Burlington Arcade, Piccadilly, London, at M. A. Stapleton's.


*Anglo-American Dental Depot.*—We copy the following proposition from the last number of the "British Journal of Dental Science," at the request, we presume, of the author, Mr. J. Ravenscroft. If a depot, such as he proposes, were established in London, it would, no doubt, extend the sale of the various materials used by dentists, manufactured in the United States. We think, therefore, it would be to the interest of manufacturers here to encourage the enterprize. EDS.

*To the Editor of the British Journal of Dental Science.*

SIR—As the British Journal is the most fitting medium to address the dental profession on matters which truly interest them, allow me to make a proposition which I conceive will fully meet the want they have felt of a veritable representation of all that is eminently good and useful among our transatlantic brethren, certain too that it proceeds from higher motives than those which merely excite prejudices. I trust that the real importance of the present subject will lawfully apologise for the intrusion it may make upon you.

A further comment would perhaps be a needless occupancy of your valuable space, but feeling assured that a liberal and educated profession will too justly appreciate the best of motives, I put it forth as bread upon the waters, in the hope that every candid mind will recognise in it a real good. I therefore beg to submit the following; viz. I propose the establishment of an *Anglo-American*, or universal *Dental Depot Company*, on a scale commensurate with the demands of the profession—the which shall truly represent all the foreign dental depots, and especially everything of worth in American dentistry to be *imported* of their respective manufacturers—and from the conviction that the best mode of placing so desirable an object on a firm basis suited to its broad intentions is through a legally organized company composed of active and silent partners, and also those of the profession who would desire to invest in it as in anything else, and that the establishment shall supply all the apparatus with small or large quantities of materials used in every description of tooth manufacture, ready for compounding by the purchaser. That any gentleman of either of the great dental bodies having any improvement in tooth manufacture, or other dental inventions, &c., which, from any inconvenience, may not be in a position to develop, shall have the privilege to suggest to any official of the company, or, at his own option, produce in the company's laboratory his improvement, invention, &c., and all such inventions shall take his naming, the object would be to give the inventor full credit. And the company shall so organize their regulations as *not* to interfere in the *least* with either of the *dental institutions*, but, as I have stated, to be truly a representative of American and other foreign dental depots, by bringing every item of value of those dental laboratories before the English dentist.

As touching the several amounts necessary to constitute shares (in the absence of any formation of the kind, and merely to convey an idea of what would be necessary to its success,) I would suggest five classes of shares, to range from *twenty-five pounds* to *one hundred pounds*, and the capital thus raised to be five thousand pounds.

Should a sufficient number of gentlemen so far approbate this proposition as to co-operate, a prospectus of the whole would be published, the arrangements completed with the great establishments, and the depot opened, so soon as circumstances would justify.

From the most fitting reasons I may only for the present receive communications by post, which shall be regarded in the strict confidence of a private correspondence. I shall therefore feel most happy to render any assistance necessary to a clearer understanding to any gentleman throughout Great Britain, who feeling disposed to support so valuable a project will favor me with a line.

J. Ravenscroft.

10 & 11 *Burlington Arcade, Piccadilly, London, at M. A. Stapleton's.*

